# Synthetic Silica Nano‐Organelles for Regulation of Cascade Reactions in Multi‐Compartmentalized Systems

**DOI:** 10.1002/anie.202113784

**Published:** 2021-12-27

**Authors:** Shuai Jiang, Lucas Caire da Silva, Tsvetomir Ivanov, Milagro Mottola, Katharina Landfester

**Affiliations:** ^1^ Max Planck Institute for Polymer Research Ackermannweg 10 55128 Mainz Germany; ^2^ Key Laboratory of Marine Drugs Chinese Ministry of Education School of Medicine and Pharmacy Ocean University of China Qingdao 266003 China; ^3^ Universidad Nacional de Córdoba CONICET, Instituto de Investigaciones Biológicas y Tecnológicas (IIBYT) Av. Vélez Sarsfield 1611, 5016 Córdoba Argentina

**Keywords:** cascade reactions, enzymatic reactions, nanoorganelles, nanoreactors, synthetic cells

## Abstract

In eukaryotic cells, enzymes are compartmentalized into specific organelles so that different reactions and processes can be performed efficiently and with a high degree of control. In this work, we show that these features can be artificially emulated in robust synthetic organelles constructed using an enzyme co‐compartmentalization strategy. We describe an in situ encapsulation approach that allows enzymes to be loaded into silica nanoreactors in well‐defined compositions. The nanoreactors can be combined into integrated systems to produce a desired reaction outcome. We used the selective enzyme co‐compartmentalization and nanoreactor integration to regulate competitive cascade reactions and to modulate the kinetics of sequential reactions involving multiple nanoreactors. Furthermore, we show that the nanoreactors can be efficiently loaded into giant polymer vesicles, resulting in multi‐compartmentalized microreactors.

## Graphical abstract

Synthetic silica nano‐organelles designed to regulate kinetics and pathway selection in enzymatic cascade reactions are reported. Like cell organelles, synthetic nano‐organelles can be combined to create integrated systems. Multicompartmentalized synthetic cells were built using synthetic nano‐organelles and giant polymeric vesicles.

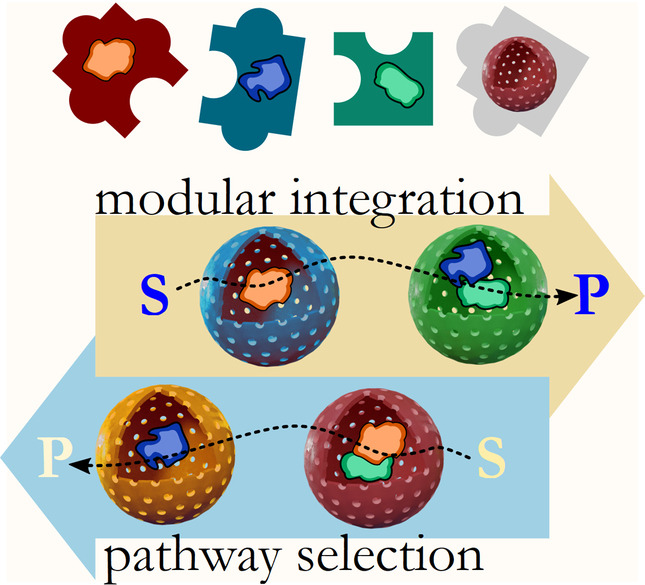

## Introduction

Compartmentalization is an essential characteristic of life because it enables the spatiotemporal control over multiple biological processes in living cells.^[1]^ Cellular compartments, such as vesicles and biomolecular condensates, provide a confined space that increases the efficiency and selectivity for enzymatic reactions and separates sensitive biological processes from harmful components present outside the compartments.^[2]^ Over the years, considerable efforts have been devoted to mimicking the compartmentalization of catalytic processes found in nature with synthetic analogues.^[3]^ These attempts have largely extended our understanding of the complex metabolic processes in living organisms, and also resulted in the development of artificial systems with the ability to mimic cellular structure and behaviour.^[4]^ Various cell‐mimetic systems based on liposomes and polymersomes have been developed.^[5]^ Sponge‐like materials and coacervate‐based compartments have also been created for this purpose.^[6]^

The encapsulation of enzymes in polymeric or lipid‐based compartments result in synthetic organelles that mimic organelles found in eukaryotic cells, such as lysosomes and peroxisomes.^[7]^ However, membrane‐based synthetic organelles usually suffer from low encapsulation efficiency and low membrane permeability, which limit their application in multi‐compartmentalized systems. Consequently, it remains a challenge to create synthetic multi‐organelle systems that, like cellular organelles, can be integrated to precisely regulate the performance and outcome of enzymatic cascade reactions.

Here, we describe a strategy for programming the outcome of enzymatic cascade reactions using nanoreactors designed as synthetic organelles. To do that, we quantitatively encapsulated enzymes in semipermeable silica nanocapsules to control the enzyme distribution and the resulting functionality of integrated multi‐organelle systems. Reaction outcomes and performance regulation was achieved by combining different nanoreactors. This strategy allowed us to (i) increase the efficiency of a three‐step sequential reaction and (ii) select distinct pathways in competitive enzymatic reactions. To demonstrate the robustness and versatility of the silica nanoreactors, we built multi‐compartmentalized micro‐reactors with a hybrid nano‐in‐micro architecture using a bottom‐up approach. The cascade reactions between different nanoreactors inside the micro‐reactors mimic the structure and basic function of natural organelles in eukaryotic cells.

## Results and Discussion

In the first step, we prepared organelle‐mimicking nanoreactors (NRs) by synthesizing core–shell silica nanocapsules with in situ encapsulated enzymes. Silica nanocapsules possess unique advantages when it comes to enzyme encapsulation efficiency because of their capsular configuration.^[8]^ A large internal cavity works as cargo reservoir, while the mesoporous silica shell provides protection and selective permeability. In addition to improved mechanical and encapsulation properties, silica nanocapsules benefit from the versatile chemistry of silica materials.

**Figure 1:**
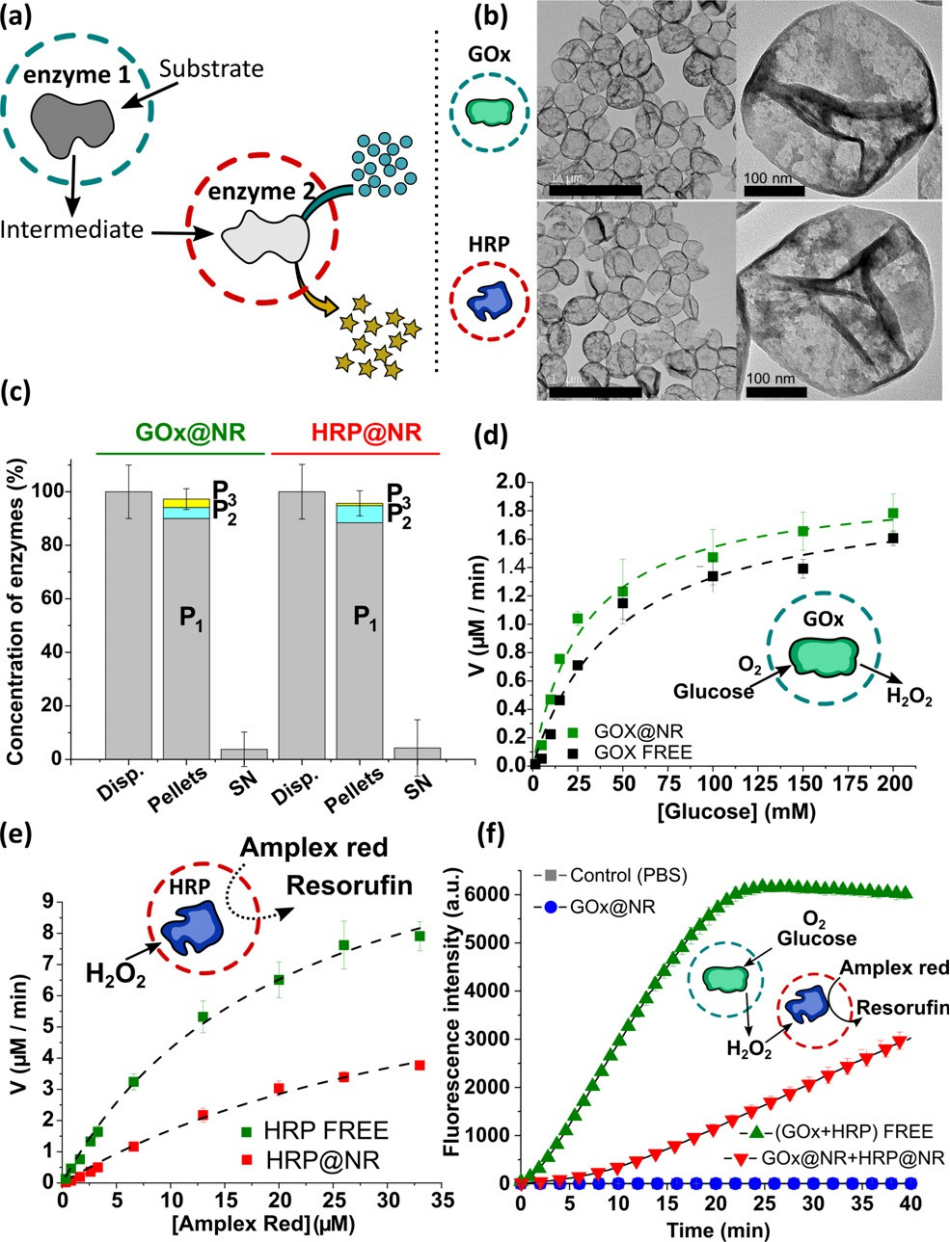
Characteristics of enzymatic nanoreactors. a) Schematic illustration of cascade reaction between individual nanoreactors (NRs). b) TEM images of GOx@NRs (top) and HRP@NRs (bottom). Scale bars=1 μm (left column) and 100 nm. c) Encapsulation efficiency (EE, %) of GOx and HRP in the NRs. The EE was determined after centrifuging the dispersions to separate encapsulated from non‐encapsulated enzymes. Disp.: original dispersion, Pellets: NRs collected after centrifugation, SN: supernatant. d–f) Michaelis–Menten kinetics of reactions involving GOx, HRP and GOx+HRP encapsulated and non‐encapsulated into NRs. Tables with 95 % confidence intervals values are shown in the Supporting Information. The NRs concentration in (d–f) was 0.25 mg mL^−1^.

Cascade reactions between individual nanoreactors were designed to mimic a multi‐step intracellular catalytic process (Figure 1 a). As a model cascade reaction, we prepared nanoreactors containing glucose oxidase (GOx) or horseradish peroxidase (HRP), labelled as GOx@NRs and HRP@NRs, respectively.

The NRs were synthesized by an inverse (water‐in‐oil) miniemulsion polymerization process (Figure S1). In short, the enzymes (GOx or HRP) were first dissolved in sodium phosphate buffer, which was then dispersed in cyclohexane by miniemulsification. The resulting enzyme‐carrying nanodroplets provided a template for the subsequent formation of a silica wall from silica precursors. The polymerization was initiated upon contact of the added precursors with the water phase in the nanodroplets. The (3‐aminopropyl)triethoxysilane precursor catalyzed the rapid condensation of tetramethoxysilane at the water‐oil interface, resulting in a porous silica shell around the nanodroplets.^[9]^ Hence, enzymes were directly encapsulated in the interior of the silica nanocapsules during the nanoreactor formation. Nanoreactors prepared in cyclohexane were then transferred to an aqueous medium with the stabilization offered by a PEG‐based surfactant, Lutensol AT50, which enhances the stability of nanoparticles in biological media.^[10]^

Dynamic light scattering (DLS, Figure S2) and transmission electron microscopy (TEM, Figure 1 b) analysis show uniform nanoreactors with the size of ca. 300 nm in diameter. The surface charge of the nanoreactors was close to neutral because of the PEG coating (Table S1). Due to the small pore size of the silica shell (Figure S3) and the relatively large enzyme size (GOx: 7.7×6.0×5.2 nm^3^; HRP: 6.2×4.3×1.2 nm^3^),^[11]^ this nanoencapsulation approach could efficiently trap the pre‐loaded enzymes. The encapsulation efficiency of enzymes in the NRs was determined by isolating the NRs from non‐encapsulated enzymes through centrifugation. The enzyme concentration in the NR‐containing pellets and in the supernatants, which contained the non‐encapsulated enzymes, were measured by BCA protein assay (Figure S4). A high encapsulation efficiency (>95 %) was obtained for both GOx and HRP (Figure 1 c). In a single nanoreactor, there was approximately 140 GOx molecules or 682 HRP molecules loaded.

The encapsulated enzymes remained active and were accessible to external reactants (Figure 1 d,e). The semipermeable silica shell, on one hand, kept the enzymes from escaping the nanoreactor. On the other hand, it allowed the transport of reactants and products across the silica wall. The enzymatic activity of GOx@NRs and HRP@NRs was measured with the Amplex^TM^ red fluorescence assay. The reaction kinetics of enzyme@NRs were compared to the results obtained from non‐encapsulated enzymes in solution (Figure 1 d,e, S5). GOx oxidizes glucose in the presence of oxygen to produce glucolactone and hydrogen peroxide (H_2_O_2_), which is a substrate for HRP. The maximum reaction velocity (*V*_max_) of encapsulated GOx was similar to the free GOx, indicating that the enzyme activity was preserved after encapsulation. The substrate affinity (*K*_m_) and the turnover number (*k*_cat_) of GOx were reduced after encapsulation. For HRP, the *k*_cat_ and the enzymatic activity were lower in the encapsulated form. The reduced enzymatic activity can be attributed to the additional diffusion barriers offered by the silica shell and the PEG layer present on the shell surface. We assume that the temporary product accumulation within the confined space, due to delayed diffusion of the products, resulted in a stronger inhibition of HRP, leading to the observed reduced turnover number.

Next, we tested a cascade reaction involving GOx@NRs and the HRP@NRs (Figure 1 f). Glucose was provided to the system and consumed by GOx@NRs to produce H_2_O_2_. The H_2_O_2_ was then used as a reactant by HRP@NRs. Oxidation of the non‐fluorescent probe Amplex^TM^ red generated a highly fluorescent molecule, resorufin. The kinetics of the cascade reactions was monitored by recording the changes in fluorescence intensity of resorufin over time (Figure 1 f). GOx@NRs, which produces H_2_O_2_, could not oxidize Amplex^TM^ red by themselves, which confirmed that both nanoreactors must be present in the system to give the final product (resorufin). The reduced kinetics shown by the NRs resulted from the additional diffusion barriers provided by the silica shell and the larger distances between the encapsulated reaction centers.

In eukaryotic cells, organelles can regulate distinct enzymatic pathways to accomplish different tasks required to keep the cell alive. Thanks to the strategic co‐compartmentalization of enzymes and other biological components, cells can accomplish complex tasks with high selectivity and efficiency.^[12]^ We now demonstrate that co‐compartmentalization of enzyme pairs in silica nano‐compartments can create complex nanoreactors. Like natural organelles, the NRs can be used to regulate the output of enzymatic cascade reactions depending on their internal composition. For this aim, we selected two types of multi‐step cascade reactions: (i) a three‐step sequential reaction and (ii) competitive reactions.

**Figure 2:**
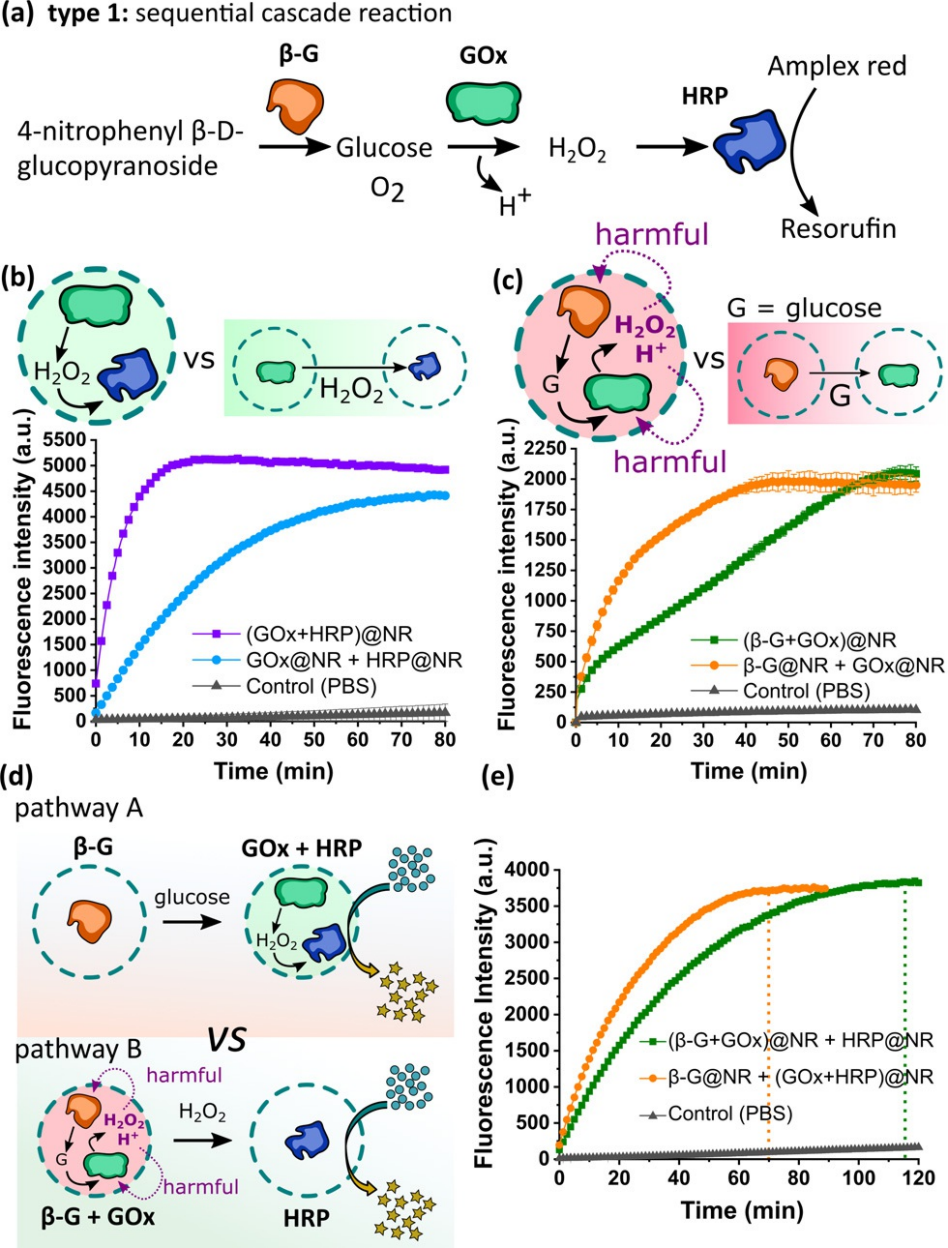
Compartmentalization in a three‐step sequential cascade reaction (type 1). a) Reaction Scheme for the sequential cascade reaction of β‐G, GOx, and HRP. b) Comparison of reaction kinetics between the co‐compartmentalized GOx and HRP and their separately loaded counterparts. A green color background represents the concentration of H_2_O_2_ in the system. c) Comparison of reaction kinetics between the co‐compartmentalized β‐G and GOx and their separately loaded counterparts. A red color background represents the concentration of H^+^ and H_2_O_2_ in the system. d,e) Scheme and reaction kinetics of the selective compartmentalization of enzymes. Pathway A: β‐G was separately loaded; GOx and HRP were co‐loaded. Pathway B: β‐G and GOx were co‐loaded; HRP was separately loaded. The error bars represent s.d. based on three identical measurements.

The type 1 cascade reaction takes three steps to be completed (Figure 2 a). In the first step, 4‐nitrophenyl β‐D‐glucopyranoside is converted to glucose by β‐glucosidase (β‐G). Glucose is then consumed by GOx to produce H_2_O_2_, which then becomes a reactant for HRP in the third step. The gluconic acid by‐product created in the second step reduces the pH in the system. A reduction of ca. 4 pH units was observed in mixtures containing GOx@NR and glucose, with the effect being dependent on the initial concentration of glucose (Figure S6). We now discuss how different encapsulation strategies lead to different reaction outcomes and performance.

First, we encapsulated GOx and HRP in the same nanoreactor and compared it with the separately encapsulated enzymes (Figure 2 b). By doing this, we were able to study the effect of the enzyme encapsulation type on the reaction kinetics. The results showed that co‐compartmentalization of GOx and HRP lead to a significantly faster reaction compared to when the enzymes are in different compartments (Figure 2 b). This is due to the confinement effect provided by the nanocontainers.

The confinement guarantees a high local concentration of the H_2_O_2_ intermediate for immediate use by the co‐encapsulated HRP. When the enzymes are in separate compartments, the reaction intermediates must diffuse from one nanocontainer to the other. This results in the dilution of the intermediate, which explains the slower reaction kinetics observed. The escape of reaction products from NRs was confirmed by the strong fluorescence intensity present in the filtrates after removal of the NRs from the reaction medium (Figure S7). Batch‐to‐batch reproducibility was demonstrated with two individually prepared samples (Figure S8). The effective co‐encapsulation of both GOx and HRP in the same NRs was proved by fluorescently labelling the enzymes and then determining the ratio of their fluorescence intensity before and after encapsulation (Figure S9).

In contrast, when we encapsulated β‐G and GOx in the same nanoreactor, a reduced reaction kinetics was observed compared with the separately encapsulated enzymes (Figure 2 c). This was caused by the accumulation of intermediates and products in the compartments. The confinement increases the local concentration of the intermediate product (glucose), which should increase the overall reaction kinetics. However, gluconic acid and H_2_O_2_ produced by GOx also accumulates internally, lowering enzyme activity due to the increased oxidizing stress and acidic environment brought about by the product accumulation (Figure 2 c). Gluconic acid and H_2_O_2_ have been reported as inhibitors of GOx.^[13]^

The previous results for a two‐enzyme system showed that the performance of the nanoreactors was determined by the types of co‐encapsulated enzymes. Co‐encapsulation of compatible pairs (GOx and HRP) enhanced the overall kinetics, while incompatible pairs (β‐G and GOx) resulted in slower kinetics. These results indicated that the performance of multi‐step cascade reactions can be regulated via the rational distribution of enzymes in different compartments via selective co‐encapsulation.

We now discuss the effect of selective enzyme compartmentalization involving three enzymes (Figure 2 d). On pathway A, β‐G was encapsulated separately, while GOx and HRP were co‐encapsulated in the same nanoreactor. On pathway B, β‐G and GOx were co‐encapsulated, with HRP loaded separately. On pathway A, the kinetics data showed that the co‐encapsulation of the two compatible enzymes (GOx and HRP) was beneficial, resulting in a more efficient reaction. As expected, the co‐encapsulation of incompatible enzymes (β‐G and GOx) on pathway B resulted in a slower kinetics. The overall cascade reaction took about 70 min to complete on pathway A. This time increased to ≈115 min on pathway B (Figure 2 e). Therefore, we were able to regulate the cascade reaction by simply rearranging the enzymes by encapsulation into distinct compartments.

**Figure 3:**
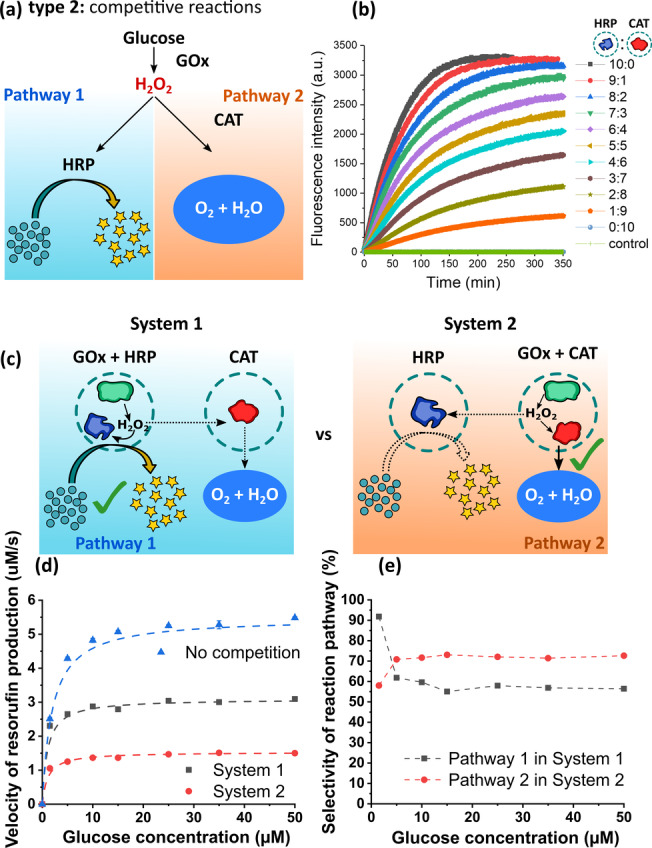
Compartmentalization in competitive reactions (type 2). a) Reaction Scheme for the competitive reactions of HRP and CAT following the reaction with GOx. b) Fluorescence intensity of reaction product resorufin in pathway 1 in the presence of different ratios of HRP@NR and CAT@NR. Control: experiment without HRP@NR and CAT@NR. c) Scheme of the selective compartmentalization of enzymes. System 1: GOx and HRP were co‐loaded; CAT was loaded separately. System 2: HRP was loaded separately; GOx and CAT were co‐loaded. d) Fluorescence intensity of reaction product resorufin from pathway 1 for system 1 and 2. A model system containing separately loaded GOx and HRP, in the absence of CAT (no competition), was used as reference (considered as 100 % of reaction via pathway 1). e) The selectivity of reaction to pathway 1 or 2 was calculated from the data shown in Figure 3 d. The reaction selectivity towards pathway 1 in system 1 was calculated by dividing the resorufin production rate of system 1 (grey line) by the control system (blue line). While the reaction selectivity towards pathway 2 in system 2 was calculated by 100 % minus the percentage obtained from dividing the resorufin production rate of system 2 (red line) by the control system (blue line). The error bars represent s.d. based on three identical measurements.

The ability to control reaction kinetics in different compartments is used by cells to select specific reaction pathways out of many possibilities. Based on this idea, we designed a pathway‐selection experiment that demonstrates that groups of nanoreactors can be programmed to select one specific pathway out of two possible choices. This is shown in the reaction type 2 (Figure 3 a), which represents a competitive cascade reaction involving three compartmentalized enzymes. First, the GOx@NR consumes glucose and produces H_2_O_2_, which becomes a substrate for two possible pathways. On pathway 1, H_2_O_2_ is consumed by HRP@NR to oxidize Amplex^TM^ red and produce resorufin. On pathway 2, the H_2_O_2_ is disproportionated by catalase (CAT@NR) to generate oxygen and water. By tuning the ratio of HRP@NR to CAT@NR, the reaction outcome can be switched between the two pathways (Figure 3 b). For example, the fluorescent signal from resorufin was zero when CAT@NR was the only active NR in the system (pathway 2 selected). When the ratio of HRP@NR to CAT@NR increased, the maximum fluorescence intensity also increased, indicating a gradual shift in selectivity towards pathway 1. Therefore, the reaction pathway can be selected by controlling the ratio of the competitive NRs in the system.

Controlling the ratio of the nanoreactors is only one way to select a given reaction pathway. Another possibility is to design nanoreactors intrinsically programmed to favour a desired reaction pathway. That was achieved through combinations of dual‐NRs and single‐NRs as shown in Figure 3 c. We co‐encapsulated GOx with either HRP (system 1) or CAT (system 2). The production of fluorescent resorufin was monitored in both systems (Figure 3 d). The results show that by this selective compartmentalization, the reaction can be programmed to favour either pathway 1 or pathway 2 (Figure 3 e). In system 1, the selectivity towards pathway 1 was over 90 % at a low concentration of glucose, and was attributed to the confinement effect that increases the local concentration of the H_2_O_2_ intermediate. As the glucose concentration increases, the selectivity to pathway 1 starts to decrease, stabilizing at about 55–60 %. We attribute the selectivity loss to the effect caused by the saturation of HRP at high H_2_O_2_ concentrations. Moreover, a higher amount of gluconic acid is expected as the glucose concentration increases, which contributes to a reduced enzyme activity due to the local acidification. System 2 led to a reaction selectivity of over 70 % for pathway 2. Interestingly, the selectivity for pathway 2 in system 2 was slightly increased at the higher concentrations of glucose. This could be attributed to the fact that the decomposition of H_2_O_2_ is an exothermic reaction,^[14]^ which leads to a higher enzyme activity due to the increased local temperature. Results from system 1 and 2 showed that we can program the outcome of cascade reactions using silica nanoreactors. The rational design and integration of enzymatic nanoreactors lead to the selection of the desired pathways.

**Figure 4:**
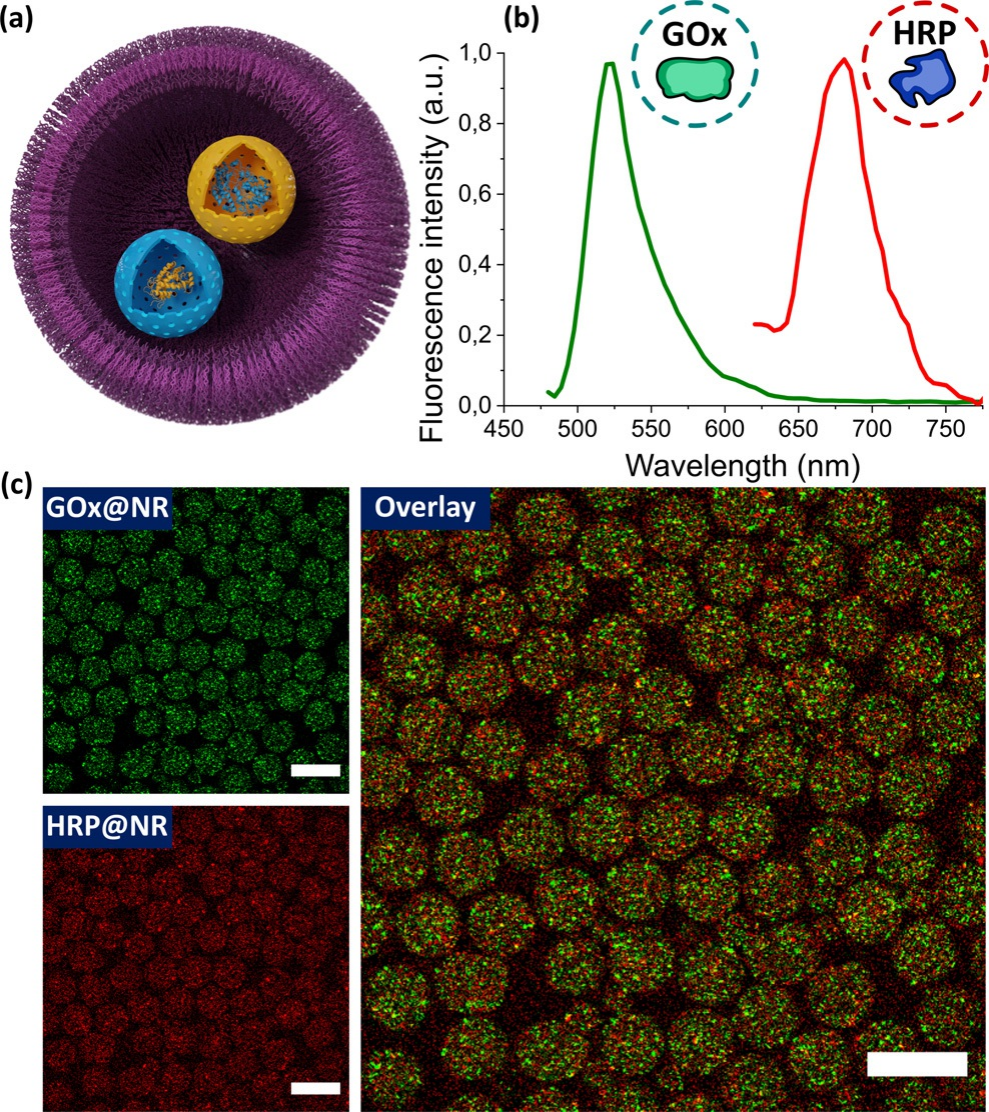
a) Scheme of a synthetic cell with nanoreactors encapsulated into a micron‐sized polymer vesicle. b) Fluorescence emission of differently labelled NRs. GOx@NRs were labelled with FITC; HRP@NRs were labelled with Cy5. c) Confocal microscopy images showing the distribution of GOx@NR and HRP@NR in the micron‐sized polymer vesicles. Green signal represents GOx@NR; red signal represents HRP@NR; the overlay image shows the co‐localization of both NRs in the vesicles. Scale bars=100 μm.

To test if NRs could be used as effective synthetic organelles for synthetic cells, we designed a simple synthetic cell containing GOx@NRs and HRP@NRs as nano‐organelles. The synthetic cells were assembled by encapsulating the NRs into giant polymer vesicles made from the block copolymer poly(butadiene)_22_‐*block*‐poly(ethylene oxide)_14_ (PB‐PEO). Amphiphilic copolymers, such as PB‐PEO, can assemble into soft membrane vesicles that are structurally like phospholipid‐based membranes, but more robust due to the higher molecular weight of the polymers.^[15]^ The polymer vesicles containing the encapsulated NRs were obtained by microfluidics (Figure S10).^[16]^ The encapsulation of nanoreactors into micron‐sized vesicles produced synthetic cells featuring the nano‐in‐micro architecture shown in Figure 4 a.

The NRs were separately labelled with Cy5 and FITC, making them easy to detect and distinguish by confocal laser scanning microscopy (CLSM). Figure 4 b shows the fluorescence emission spectra of the labelled NRs. Figure 4 c shows the CLSM images of the synthetic cells carrying the NRs. The NRs were homogeneously distributed in the vesicles. Each vesicle contained about 10^4^ randomly distributed NRs. One advantage of using silica nanoreactors is that the robust silica shell protects the enzymes during the vesicle preparation and later manipulations. This extra stability allows the assembly of robust synthetic cells that can last for days after their preparation. In addition, silica nanoreactors are naturally porous. Therefore, there is no need to account for the lower permeability usually observed when amphiphilic polymers and lipids are used to create soft NRs.^[17]^ Substrates and products can easily reach and leave the pores of the silica NRs, facilitating the communication between the NRs inside the synthetic cells.

**Figure 5:**
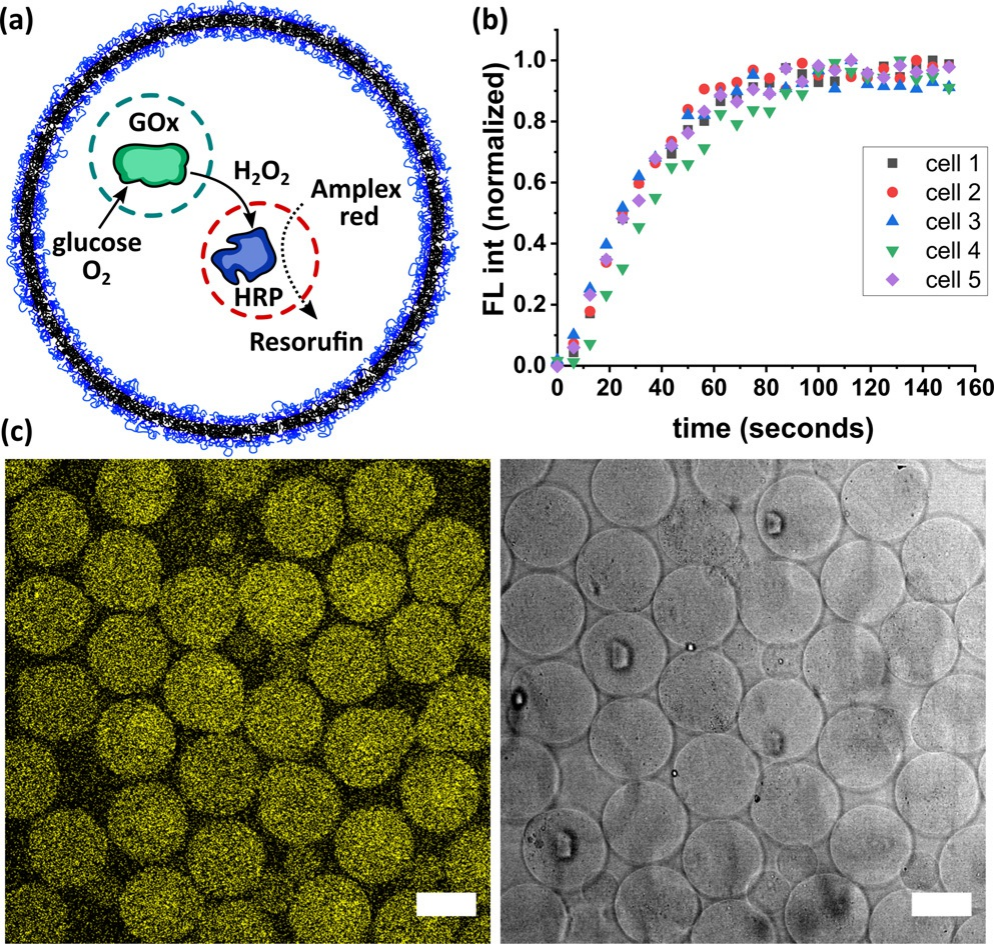
Cascade reaction in a synthetic cell. a) Schematic illustration of the cascade reaction promoted by GOx@NR and HRP@NR in a synthetic cell. b) Fluorescence intensity of the product (resorufin) over time obtained by confocal microscopy for 5 individual synthetic cells. Time step size: 6.5 seconds. c) Confocal microscopy images of the reaction end points. Left: resorufin fluorescence. Right: bright field image. Scale bars=50 μm.

To demonstrate the activity of the synthetic cells, we reproduced the cascade reaction between GOx@NR and HRP@NR inside the vesicles (Figure 5 a). Addition of Amplex^TM^ red into the external aqueous medium resulted in the formation of the product (resorufin) inside the vesicles confirming that the activity of the NRs is not affected by the encapsulation into the vesicles (Figure 5 b).

Amplex^TM^ red can cross the polymer membrane because of its hydrophobic nature. Once the molecules reach the interior of the vesicles, they must cross the porous silica wall of the NRs before the oxidation to resorufin can take place. Figure 5 b shows the evolution of fluorescence intensity from resorufin in the vesicles. The data was acquired by confocal laser microscopy at regular intervals (6.5 s steps). The signal intensity sharply increases between 20 and 70 seconds, eventually reaching a plateau that marks the end of the reaction. The initial reaction rate and subsequent behaviour was consistent among the different vesicles, indicating a high homogeneity in terms of vesicle size and composition. Figure 5 c shows the emission intensity of the product confined inside the synthetic cells at the reaction end point. The images show that the vesicle membrane retained its integrity during the course of the reaction.

## Conclusion

In summary, we described a strategy for the creation of modular biomimetic synthetic nano‐organelles that can be combined to perform different tasks. Through an in situ encapsulation approach, we were able to program the outcome of enzymatic cascade reactions and enhance reaction efficiency by selectively compartmentalizing the enzymes. Silica nanoreactors have a high colloidal stability which facilitated their incorporation into polymer vesicles, resulting in nano‐in‐micro multi‐compartmentalized synthetic cells. Robust and scalable strategies for the regulation of multi‐compartmentalized cascade reactions are required for the development of the next generation of biomimetic system. The silica nanoreactors described in this work offer an alternative to biological and hybrid confined systems that suffer from low colloidal stability and limited permeability.^[18]^

## Conflict of interest

The authors declare no conflict of interest.

## Supporting information

As a service to our authors and readers, this journal provides supporting information supplied by the authors. Such materials are peer reviewed and may be re‐organized for online delivery, but are not copy‐edited or typeset. Technical support issues arising from supporting information (other than missing files) should be addressed to the authors.

Supporting Information
