# Supplementary material for: Synthetic Silica Nano‐Organelles for Regulation of Cascade Reactions in Multi‐Compartmentalized Systems
Source: Angew Chem Int Ed Engl. 2021 Dec 27;61(6):e202113784. doi: 10.1002/anie.202113784 (PMC9306467; doi:10.1002/anie.202113784)
Supplement: Supplementary file 1 — Supporting Information [file ANIE-61-0-s001.pdf]

Angewandte Chemie
Eine Zeitschrift der Gesellschaft Deutscher Chemiker

Supporting Information

Synthetic Silica Nano-Organelles for Regulation of Cascade Reactions in Multi-Compartmentalized Systems

*Shuai Jiang[+], Lucas Caire da Silva[+], Tsvetomir Ivanov, Milagro Mottola, and Katharina Landfester**

anie_202113784_sm_miscellaneous_information.pdf

Supporting Information

Table of Contents

1. EXPERIMENTAL SECTION 2
1.1 Materials 2
1.1.1 Nanoreactors 2
1.1.2 Polymer vesicles 2
1.2 Synthesis of enzyme-loaded silica nanocapsules as organelle-mimetic modules 2
1.3 Characterization of silica nanocapsules 3
1.4 Quantification of enzyme encapsulation in nanocapsules 3
1.4.1 Encapsulation efficiency of enzymes 3
1.4.2 Fluorescent labeling of enzymes 4
1.5 Enzyme activity test 4
1.5.1 Nanoreactor permeability test 5
1.5.2 Batch-to-batch reproducibility 5
1.6 Microfluidics 6
1.6.1 Photolithography 6
1.6.2 PDMS chip production 6
1.6.3 Chip coating 7
1.6.4. Fluid compositions 8
1.6.5 Vesicle production 8
1.6.6 Microscopy 9
2. RESULTS 9
3. REFERENCES 16

1. EXPERIMENTAL SECTION

1.1 Materials

1.1.1 Nanoreactors

Cyclohexane (≥99, VWR Chemicals), polyglycerol polyricinoleate (GRINDSTED®PGPR 90 KOSHER, DANISCO), tetramethyl orthosilicate (TMOS, 99%, Sigma-Aldrich), (3-aminopropyl)triethoxysilane (APTES, 99%, Sigma-Aldrich), and Lutensol AT50 (powder, BASF) were used as received. Glucose oxidase from *Aspergillus niger* (GOD, Type X-S, lyophilized powder, 100,000-250,000 units/g solid, G7141-50KU) and peroxidase from horseradish (HRP, Type II, essentially salt-free, lyophilized powder, 150-250 units/mg solid, P8250-50KU) were supplied by Sigma-Aldrich. Ultrapure water was used in all experiments.

1.1.2 Polymer vesicles

Sucrose, D-(+)-glucose, D-(−)-fructose, Amplex^{TM} red, natriumhydroxide, oleyl alcohol (80-85% tech. grade), dimethyl sulfoxide and Sylgard 184 elastomer kit were all purchased from Sigma Aldrich. The block polymer poly(butadiene)-*block*-poly(ethylene oxide) (PB-PEO) was obtained from Polymer Source Inc (Canada). Syringes (1 ml) were purchased from Henke-Sass, Wolf GmbH, while 0.55 mm hypodermic needles and 30 M PTFE microbore tubing were purchased from Fisher Scientific GmbH. Ultrapure water was used in all experiments.

1.2 Synthesis of enzyme-loaded silica nanocapsules as organelle-mimetic modules

Silica nanocapsules were synthesized by using an inverse (water-in-oil) miniemulsion technique (see Figure S1). Enzymes (*e.g.* GOx or HRP) were directly encapsulated in the aqueous core of the nanocapsules during capsule formation.

Specifically, an aqueous phase and an oil phase were prepared separately. The enzyme was pre-dissolved at a concentration of 5 mg mL^{-1} in the aqueous phase (sodium phosphate buffer, 10 mM, pH 7.4). The oil phase was composed of a solution of 26.7 mg mL^{-1} PGPR in cyclohexane. For preparing emulsions, 30 mL oil solution was poured into the vial containing 1 mL aqueous phase under magnetic stirring at 1000 rpm. The oil-water mixture was homogenized with a T25 Ultra-Turrax at 13,000 rpm for 1 min. The emulsion was then processed in a microfluidizer (LM10, Microfluidics Corporation) cooled with water. The

microfluidizer is equipped with an air-driven intensifier pump that supplies the desired pressure to the emulsion stream. The processed emulsions were collected from the outlet flow and were fed again to the inlet reservoir. Each emulsion was processed for two cycles of microfluidization. After the emulsification, a mixture of 335 μL of TMOS and 100 μL of APTES were added to the emulsion in 10 seconds under stirring at 1000 rpm. Samples were kept stirring at room temperature for another 12 h before the sol-gel reactions were completely finished.

The synthesized nanocapsules in cyclohexane phase were then transferred to an aqueous medium. For this purpose, 6 mL of Lutensol AT50 solution (0.3 wt% in water) was prepared and placed in a sonication bath. Next, 0.6 mL of nanocapsule-cyclohexane dispersion was added dropwise to the Lutensol solution in 5 min under shaking. The mixture was kept stirring in an open flask (without lid) at 1000 rpm and room temperature for 24 h to evaporate the cyclohexane. The samples in aqueous medium were then washed 3 times with water by centrifuging at 1660 g for 20 min at 10 °C. Supernatants from the centrifugation step were discarded and fresh water was added. The dispersions were then homogenized by repetitive pipetting.

1.3 Characterization of silica nanocapsules

The average size and size distribution of nanocapsules were measured by dynamic light scattering (DLS) at 25 °C on a Nicomp 380 submicron particle sizer (Nicomp Particle Sizing Systems, USA) at a fixed scattering angle of 90°. Zeta potential measurements were performed in 10^{-3} M potassium chloride solution at pH 6.8 and 25 °C with a Malvern Zeta sizer (Malvern Instruments, UK). Solid content of the capsule dispersion was measured gravimetrically. The morphology of nanocapsules was examined with a Jeol 1400 (Jeol Ltd, Tokyo, Japan) transmission electron microscope (TEM) operating at an accelerating voltage of 120 kV. Samples for TEM were prepared by casting the diluted dispersions (solid content: ~0.01 wt%) on carbon layer-coated copper grids.

1.4 Quantification of enzyme encapsulation in nanocapsules

1.4.1 Encapsulation efficiency of enzymes

Encapsulation efficiency of enzymes in the nanocapsules was determined by separating the capsules from non-encapsulated enzymes in the dispersion by centrifugation. Firstly, 1 mL of nanocapsule dispersion (solid content = 1.0 wt%) was centrifuged at 1760 g for 40 min at 10 °C. The pellet was collected, and the supernatant (SN) was transferred to a new tube for another centrifugation step. The centrifugation was repeated for three times. The enzyme contents in the three pellets and in the final supernatant were determined by using BCA (bicinchoninic acid) protein assay (**Figure S4**). 5 μL of the sample was incubated with 150 μL of BCA working reagent at 37 °C for 30 min. The absorbance at 562 nm was recorded by plate reader (TECAN M1000). Because of the silica absorption at this wavelength, the final enzyme content was determined by subtracting the total absorption with the absorption of empty silica NRs as the control sample. The encapsulation efficiency was calculated by using the following equation: $\%EE = (P_1+P_2+P_3)/(SN_3+P_1+P_2+P_3)*100\%$, where P_n represents the pellet collected at centrifugation step (n), and SN_n represents the supernatant obtained from the centrifugation step (n).

1.4.2 Fluorescent labeling of enzymes

Firstly, a stock solution of BDP FL-NHS ester or Cy5-NHS ester in DMSO was prepared. Enzymes (GOx or HRP) were dissolved in 0.1 M sodium bicarbonate solution at a concentration of 10 mg/mL. The dye solution was mixed with the enzyme solution at the volume ratio of 1:9. The final molar ratio of dye to the enzymes is 8:1. The mixture was stirred at 1000 rpm for 3 min and then incubated at 25 °C for 4 h. Afterwards, the samples were purified by using a gel-filtration column (Zeba™ Spin Desalting Columns, 7K MWCO, 10 mL; Thermo Fisher 89893). Before use, the column was cleaned three times with buffer by centrifugation at 1000g for 2 min. The labelled samples were then placed in the column and centrifugated at 1000g for 2 min. The purified samples were redispersed in buffer solution and collected in a new vial for further use of preparing nanocapsules.

1.5 Enzyme activity test

Amplex™ Red assay was used to determine the activity of encapsulated enzymes. For GOx activity test, a reaction cocktail composed of 940 μL of sodium phosphate buffer, 20 μL of glucose solution (100 mM), 10 μL of HRP solution (300 unit mL^{-1}), and 3.3 μL of Amplex Red solution (1.0 mM) was prepared freshly before use. The reaction cocktail for HRP activity test was composed of 1 mL of sodium phosphate buffer, 3.3 μL of 0.35 wt% H_2O_2 solution, and

3.3 μL of Amplex Red solution (1.0 mM). In a single well of 96-well microplate, 2.5 μL of the enzyme sample was added and followed by pouring 100 μL of the above reaction cocktail. The fluorescence emission of resorufin product at 595 nm was measured every 15 s using a TECAN M1000 microplate reader. A standard curve of fluorescence intensity of resorufin was included as **Figure S11** in the supporting information.

For Michaelis−Menten kinetics for GOx@NRs and HRP@NRs, the concentration of glucose and Amplex red were varied, respectively. The values of Michaelis constant (K_M) and maximal specific activity (S_{max}) were determined by fitting a hyperbolic equation to experimental data by computer-aided nonlinear regression analysis. The sequential and competitive cascade reactions were performed with same amounts of specific enzymes but in different (co-)encapsulation strategies as shown in Figure 2 and 3.

1.5.1 Nanoreactor permeability test

The passive diffusion of small water soluble molecules was demonstrated by fluorescence intensity measurements performed on a TECAN M1000 plate reader. A reaction cocktail was prepared with following composition: 940 µl PBS buffer + 20 µl Glucose + 3.3 Amplex Red. After homogenization, 100 µl of the reaction cocktail was placed in separate wells. Then 2.5 µl of nanocapsule dispersion was added to each well to start the reaction. The following experimental set up was used: 555 nm excitation, 595 nm emission. A kinetic cycle was performed for 1 hour with measurements taken every 15 seconds. The results are shown in **Figure S7**.

Solution Composition:

Solution	Concentration
PBS buffer	10 mM
Glucose in deionized water	10 mM
Amplex Red in DMSO	1 mM
HRP and GOx Nanocapsules in water	ca. 0.24 g/L*

* solid content of stock dispersion: 0.97 wt%

1.5.2 Batch-to-batch reproducibility

A reaction cocktail was prepared as a stock solution in the following composition: 940 µl PBS buffer + 20 µl Glucose + 3.3 Amplex Red. After homogenization, 100 µl of the reaction cocktail was placed in each well. Then either 2.5 µl of HRP and GOx nanocapsules or 1.25 µl HRP nanocapsules + 1.25 GOx nanocapsules were added to each well to start the reaction. The

measurements were performed using new and distinct batches of nanocapsules. The following experimental set up was used: 555 nm excitation, 595 nm emission. A kinetic cycle was performed for 1 hour with measurements taken every 15 seconds. The results are shown in **Figure S8**.

1.6 Microfluidics

1.6.1 Photolithography

Photolithography is the process of transferring geometric shapes on a mask to the surface of a silicon wafer. The steps involved in the photolithographic process were wafer cleaning; barrier layer formation; photoresist application; soft baking; mask alignment; exposure and development; and hard baking. In the first step, the wafers were chemically cleaned to remove particulate matter on the surface as well as any traces of organic, ionic, and metallic impurities. Then the wafers were pre-backed on a hotplate at 200 °C for 5 minutes. Then, the wafers were spin-coated with photoresist SU-83050 at 2200 rpm for 30 seconds to achieve a height of approximately 70 µm and with photoresist SU-83030 at 1800 rpm for 30 seconds again to achieve a height of 40 µm. Then the wafers were pre-baked on a hotplate for 1 minute at 65 °C, and soft-baked for 20 minutes at 95 °C and in the end for cooling down baked again at 65 °C for 1 minute. Soft baking is the step during which almost all of the solvents are removed from the photoresist coating. The wafer and the photoresist were then aligned with a film mask containing the chip designs. Once the mask had been accurately aligned with the pattern on the wafer's surface, the photoresist is exposed through the pattern on the mask with a high intensity ultraviolet light for 5 seconds. The wafer and the UV-treated photoresist were post-baked on a hotplate for 1 minute at 65 °C, then baked at 95 °C and again for 1 minute at 65 °C. Hard-baking is the final step in the photolithographic process. This step is necessary in order to harden the photoresist and improve adhesion of the photoresist to the wafer surface. The unexposed photoresist was dissolved by soaking the wafer in a developer for 10 minutes to 15 minutes. The finished wafer was washed with isopropanol and dried with nitrogen. The wafer was inspected under a microscope and the height was measure on the 4 poles of the wafer.

1.6.2 PDMS chip production

For the production of microfluidic chips, the silicon wafers with the channel molds were placed in a glass dish and covered with from 5-10 mm high mixture of liquid PDMS and curing agent in a 9:1 ration (for 1 glass dish around 40 mg of mixture was needed). The glass dish with the wafer and the liquid mixed PDMS and curing agent solution was degassed via a conventional vacuum pump and chamber until all air bubbles disappeared. The wafers were then placed at 80 °C for at least 2 hours in order to improve molecular cross-linking. Once hardened, individual PDMS-replicas were cut out using a scalpel. The three inlet channels and the outer channel are pierced with a syringe needle to create a connection between the channels and the later outside. The side of the replica with the channels was then covered with tape until the activation in the plasma cleaner to protect in from dust particles and to remove dirt from the surface. A large cover glass slide is thoroughly cleaned with 70 % ethanol. Then the glass slide is activated together with the PDMS-replica (channels from the upper side in the device) in a plasma cleaner (Diener Electronic Plasma-Surface-Technology, Model Femto) for exactly 30 seconds at the 30% power which equals to 30 W. The activated glass slide was therefore pressed carefully on the activated side of the PDMS-replica with the microfluidic channels and again baked at 80 °C for at least 90 minutes (excluding the baking time after coating) to improve the creation of the covalent bond. Finished chips were carefully stored in a glass dish protected from dust particles and dirt. Before usage the chips were inspected under microscope for irregularities or dirt inside the channels.

1.6.3 Chip coating

The chip coating is required in double emulsion chips to enable a stable emulsion at the second junction and prevent the middle fluid from sticking to the otherwise hydrophobic walls of the outlet channel. This coating method required the chips to be coated straight after activation. First a solution of 1 wt % of PVA in distilled water is prepared and filtered. To avoid undesired coating, the inner and middle fluid inlet holes were covered with tape and slightly cut open to allow for a weak air influx. The solution is then sucked with a conventional vacuum pump via the outlet fluid inlet hole through the outer fluid channel to the chip outlet for approx. 10 minutes or alternatively until 1mL PVA solution had flowed through (usually the last few µl are left to prevent dust particles entering the channels). Clogging of the channels by excess PVA solution is prevented by sucking air for 1 minute after coating. The coated chips are then baked at 120 °C for at least 30 minutes and the baked additionally 1 and a half hours at 80°C. After production, the coating of the outer channel is inspected on a microscope.

1.6.4. Fluid compositions

Inner and outer solutions were prepared fresh from stock solutions for all experiments and not used longer than one week. The respective stock solutions were not older than one week either and were prepared in ddH_2O. Sucrose, fructose and glucose were prepared at a concentration of 300 mM and their pH level was measured and maintained before every experiment in the range of 7-9. 1 M NaOH solution was used for correction of the pH value of the stock solutions. The middle solution consisted of 10 mg/mL of PB_{22}-PEO_{14} in oleyl alcohol as stock solution. 1 mM Amplex red in DMSO was prepared as a stock solution for initiation of the resorufin production reaction.

Fluid	Composition	Final concentrations
Inner	300 µL of sucrose in H_2O (1M)<br>50 µL of GOx dispersion in H_2O (12.6 mg mL^{-1})<br>50 µL of HRP dispersion in H_2O (10.9 mg ml^{-1})<br>600 µL H_2O	Sucrose, 300 mM<br>GOx, 0.63 mg mL^{-1}<br>HRP, 0.55 mg mL^{-1}
Middle	10 mg of PB_{22}-PEO_{14}<br>1 mL of oleyl alcohol	PB_{22}-PEO_{14}, 10 mg mL^{-1}
Outer	300 µL of sucrose in H_2O (1M)<br>700 µL H_2O	Sucrose, 300 mM

1.6.5 Vesicle production

The solutions created according to the previous section are sucked into 1 ml plastic syringes and connected via a needle and tubing to the inlets of the microfluidic chip. The syringes and tubing are then checked for air bubbles inside, and if there are any the chip was disconnected and the bubbles are manually driven out. Then a piece of tubing is used to connect the outlet of the chip to an Eppendorf tube. The syringes were fixed on the microfluidic pump system and the chip was placed under a microscope lens. Stable double emulsions were generated by adjusting the flow rates of outer, middle and inner fluid stepwise. With the chip fixed on the microscope, was initiated by applying pump rates of 100 µl/h for the inner and middle fluid. When the fluids reached the chip, the outer fluid was initiated with rate of 200 µl/h. If poor coating of the channels is identified or presence of dirt that could not be removed by increasing the flow rates in the affected channels, the chip had to be changed. Once the microfluidic chip

is successfully initiated, the flow rates are adjusted accordingly to the desired values of 40 µl/h for the inner and middle phases and 400 µl/h for the outer phase. The vesicles are discarded until the behaviour of the double emulsion at the second junction have stabilized. Once the equilibrium was reached the system composition remained unchanged for the duration of the experiment. The vesicles were collected via a tube in an external vessel for further analysis. For microscopy, the vesicle dispersion was transferred to an aqueous fructose solution (300 mM).

1.6.6 Microscopy

Confocal laser scanning microscopy (CLSM) images and videos were acquired using a Leica TCS SP5 II system with four solid state lasers Argon, DPPS 561 nm, HeNe 594 and HeNe 633 nm excitation wavelengths. The 561 nm laser with excitation 580 nm and emission 620 nm was used for visualization of the FITC dyed nanocapsules and resorufin, which is presented in green fluorescence. While 633 laser with excitation 650 nm and emission 690 nm was used for visualization of CY5 dye in the nanocapsules, which is presented in red fluorescence. The bright field scanning utilized grey as the main background colour. The corresponding LAS X Software allowed for creation and manipulation of image capturing. Images were acquired using immersion objectives for 25.0x0.95 water and 63x1.20 water UV magnification.

2. RESULTS

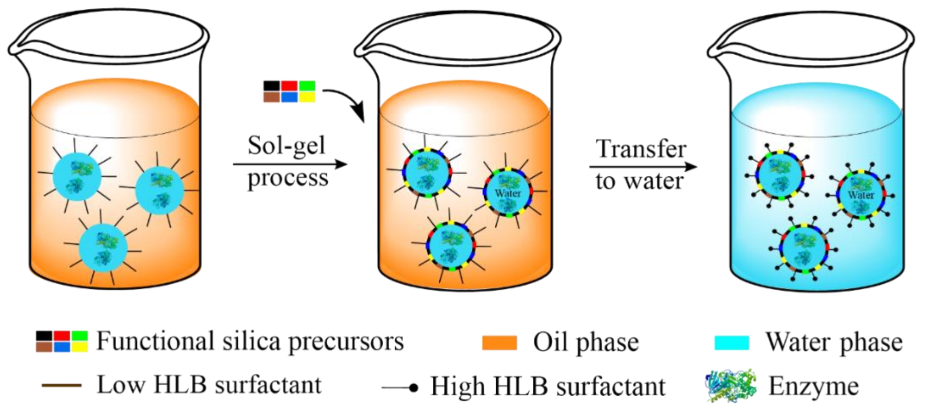

Figure S1. Schematic illustration of the preparation of silica nanoreactors with in-situ loaded enzymes by using an inverse (water-in-oil) miniemulsion polymerization process.

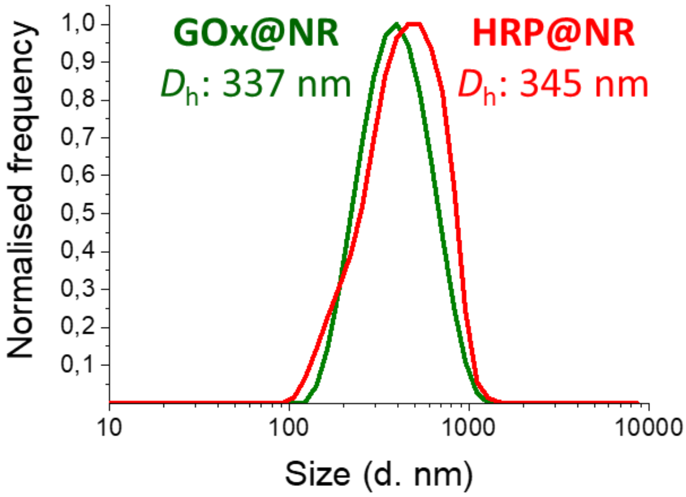

Figure S2. Size distribution of nanoreactors GOx@NRs and HRP@NRs measured by dynamic light scattering.

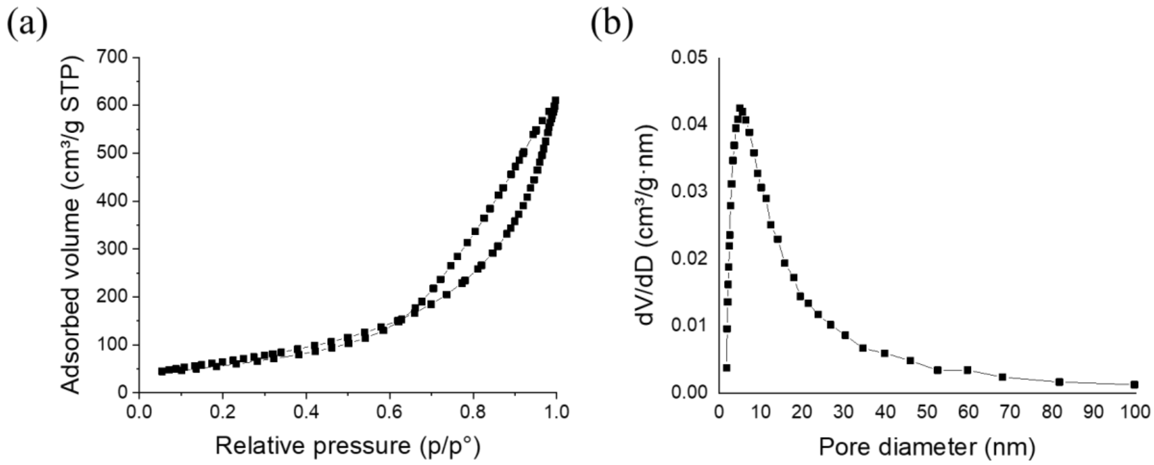

Figure S3. (a) N_2 adsorption–desorption isotherms, (b) pore size distribution curve of the silica nanoreactors. The samples were prepared by freeze-drying an aqueous dispersion of nanocapsules.

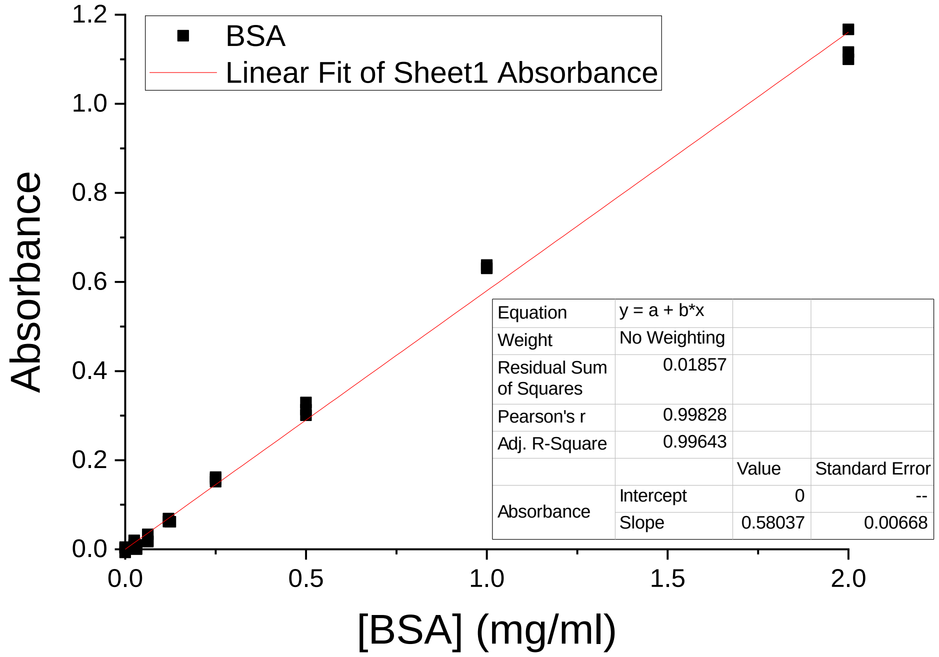

Figure S4. Calibration curve for protein quantification by BCA assay. Bovine serum albumin (BSA) was used as the standard with concentration ranging from 0 to 2.0 mg/mL.

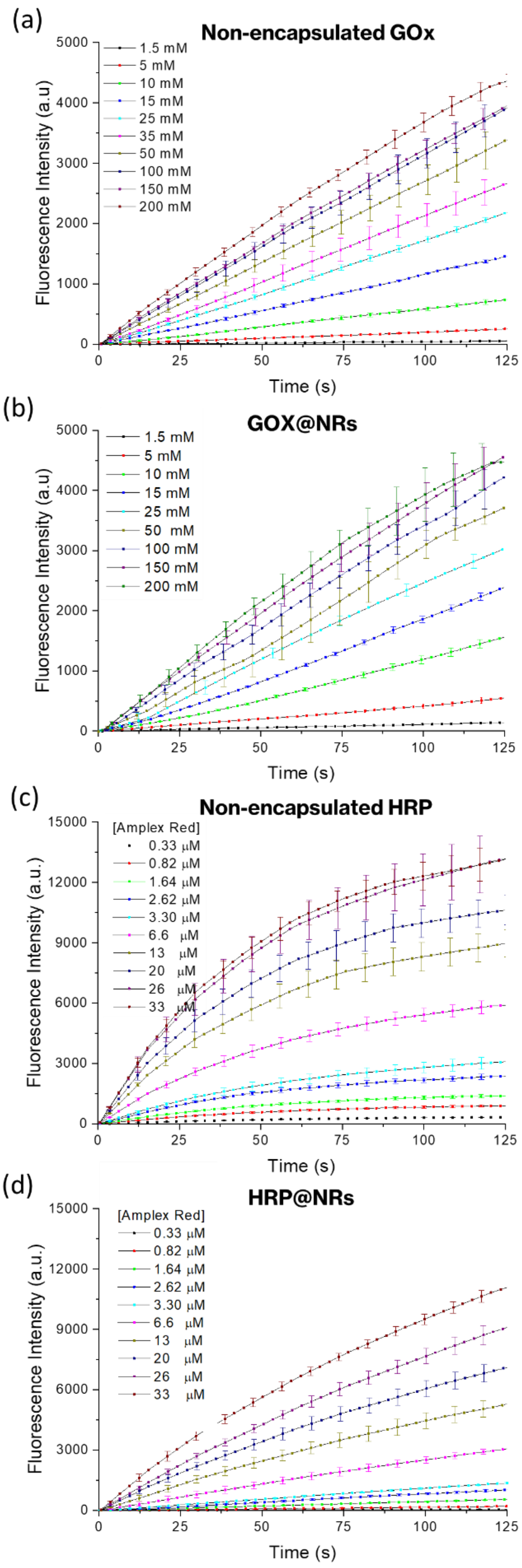

Figure S5. Raw reaction curves with varied substrate concentrations for the kinetics characterization of (a) non-encapsulated GOx, (b) GOx@NRs, (c) non-encapsulated HRP, and (d) HRP@NRs.

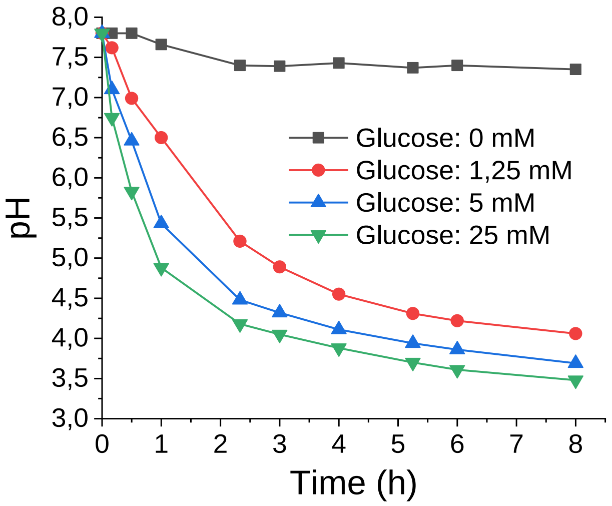

Figure S6. pH value of the solutions after incubation of GOx@NRs (1 mg/mL) with varied concentrations of glucose.

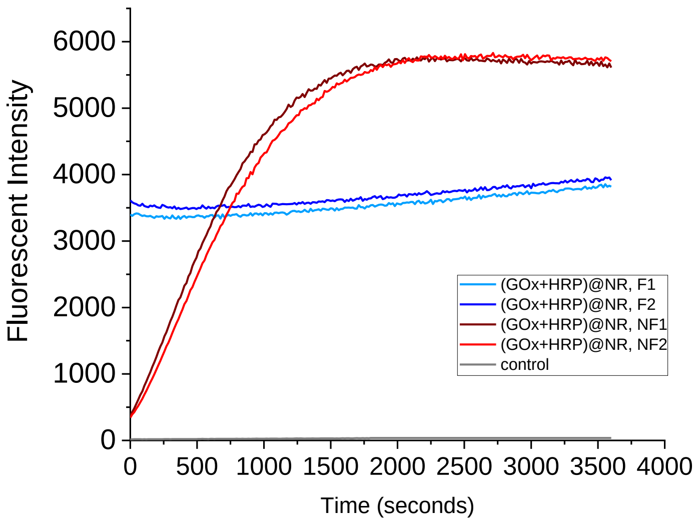

Figure S7. Fluorescence intensity measurements of resorufin production by (GOx+HRP)@NRs without removal of the NRs (non-filtered, NF) and after removal of the NRs (filtered, F). Comparison between NF and F proves that the product can diffuse out of the NRs. Samples were measured using two distinct batches of nanoreactors. The signals from F1 and F2 in the plot correspond to the amount of resorufin produced up to the moment when the NRs were removed. Since no reaction could proceed without the NRs, the signals remained constant. Additionally, the signals were significantly higher than the background signal, indicating that the product (resorufin) must have left the interior of the porous NRs. In the NF samples, the reaction proceeded as expected, eventually reaching a plateau after the substrates were consumed.

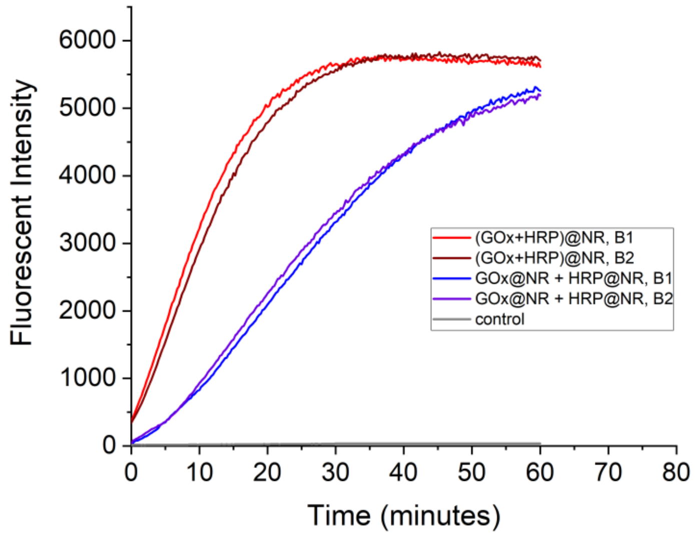

Figure S8. Batch-to-batch reproducibility of resorufin production in two distinct configurations. B1, B2: batches 1 and 2.

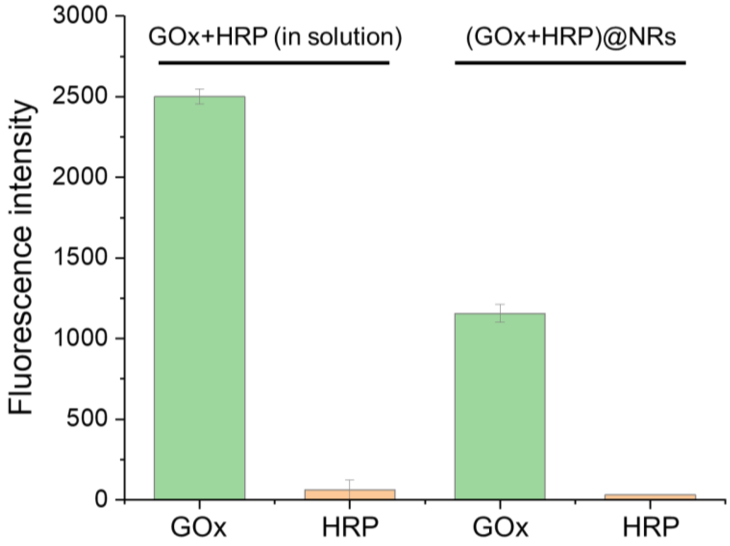

Figure S9. Fluorescence intensity of BDP FL-labeled GOx and Cy5-labelled HRP: Mixed in solution *vs* Co-encapsulated in the same nanoreactors. Excitation wavelengths of BDP FL and Cy5 were 488 nm and 638 nm, respectively. The BDP FL-labeled GOx is brighter than the Cy5-labeled HRP although at the same molar ratio of dye due to the high quantum yield of the former. The co-encapsulation of both enzymes in the same nanoreactor was determined by comparing the ratio of fluorescence intensity (FI) of GOx-BDP and HRP-Cy5 mixed in solution with the ratio of their FI after loading in the nanoreactors. FI(GOx)/FI(HRP) mixed in solution

= 39.7, FI(GOx)/FI(HRP) loaded in nanoreactor = 37.3. The close FI ratios of the two enzymes in solution and in nanoreactors prove their efficient co-encapsulation at the input stoichiometry.

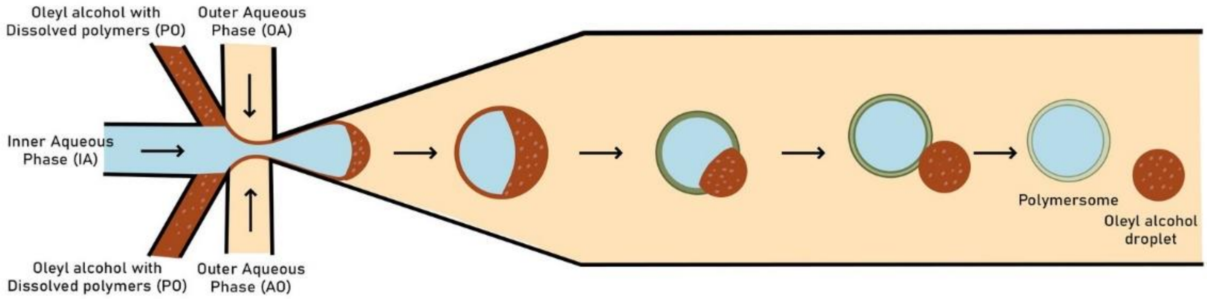

Figure S10. Scheme of the vesicle production using microfluidics.

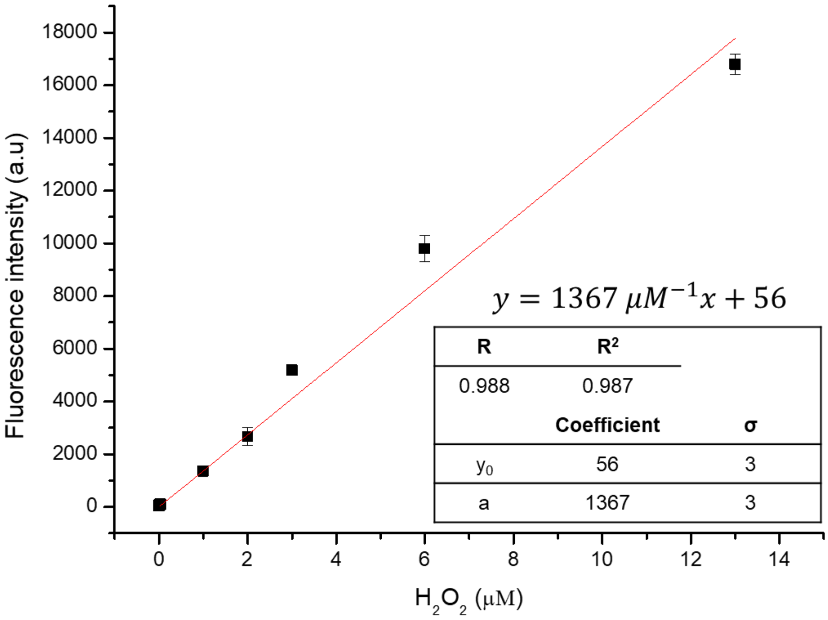

Figure S11. Standard curve of fluorescence intensity of resorufin vs. [H_2O_2].

Table S1.

	Sample ID	D_h (nm)	Zeta potential (mV)
GOx@NR	SJP131b-FITC	337 ± 81	-4.3
HRP@NR	SJP131c-Cy5	345 ± 76	-2.6

Table S2. Michaelis-Menten kinetics of GOx and GOx@NR from Figure 1d.

Sample	Vmax (μM/min)	K_m (mM)	k_{cat} (s^{-1})	k_{cat}/K_m
GOX FREE	2.0 ± 0.1	47 ± 10	46 ± 3	1.0 ± 0.2
GOX@NR	2.0 ± 0.1	29 ± 4	48 ± 1	1.6 ± 0.1

Table S3. Michaelis-Menten kinetics of HRP and HRP@NR from Figure 1e.

Sample	Vmax (μM/min)	K_m (μM)	k_{cat} (s^{-1})	k_{cat}/K_m
HRP FREE	13 ± 2	21 ± 2	81 ± 1	4 ± 1
HRP@NR	9 ± 2	42 ± 10	56 ± 3	1.5 ± 0.5

3. REFERENCES

(1) Fung, B. M.; Khitrin, A. K.; Ermolaev, K. An Improved Broadband Decoupling Sequence for Liquid Crystals and Solids. *J. Magn. Reson.* **2000**, *142* (1), 97–101.

(2) Marsmann, H. C.; Raml, W.; Hengge, E. 29Si NMR Measurements on Polysilanes. 2. Isotetrasilanes. *Zeitschrift für Naturforsch. B* **1980**, *35* (12), 1541–1547.

(3) Massiot, D.; Fayon, F.; Capron, M.; King, I.; Le Calvé, S.; Alonso, B.; Durand, J.-O.; Bujoli, B.; Gan, Z.; Hoatson, G. Modelling One- and Two-Dimensional Solid-State NMR Spectra. *Magn. Reson. Chem.* **2002**, *40* (1), 70–76.
